# Structure Dependence of Poisson’s Ratio in Cesium Silicate and Borate Glasses

**DOI:** 10.3390/ma13122837

**Published:** 2020-06-24

**Authors:** Martin B. Østergaard, Mikkel S. Bødker, Morten M. Smedskjaer

**Affiliations:** Department of Chemistry and Bioscience, Aalborg University, 9220 Aalborg East, Denmark; mbo@bio.aau.dk (M.B.Ø.); msb@bio.aau.dk (M.S.B.)

**Keywords:** oxide glasses, Poisson’s ratio, network connectivity

## Abstract

In glass materials, Poisson’s ratio (ν) has been proposed to be correlated with a variety of features, including atomic packing density (*C*_g_), liquid fragility (*m*), and network connectivity. To further investigate these correlations in oxide glasses, here, we study cesium borate and cesium silicate glasses with varying modifier/former ratio given the difference in network former coordination and because cesium results in relatively high *ν* compared to the smaller alkali modifiers. Within the binary glass series, we find positive correlations between *ν* on one hand and *m* and *C*_g_ on the other hand. The network former is found to greatly influence the correlation between *ν* and the number of bridging oxygens (*n*_BO_), with a negative correlation for silicate glasses and positive correlation for borate glasses. An analysis based on topological constraint theory shows that this difference cannot be explained by the effect of superstructural units on the network connectivity in lithium borate glasses. Considering a wider range of oxide glasses from the literature, we find that *ν* generally decreases with increasing network connectivity, but with notable exceptions for heavy alkali borate glasses and calcium alumino tectosilicate glasses.

## 1. Introduction

Oxide glasses are well-known for their brittle fracture behavior, limiting current and emerging applications [1,2,3,4]. The deformation behavior of isotropic glasses, even beyond the elastic limit, has been proposed to be closely related to Poisson’s ratio (*ν*), which is defined as the negative ratio of the transverse strain relative to the longitudinal strain in the elastic loading direction. For example, glasses with low *ν* are generally more prone to undergo a high degree of densification during indentation [5]—we here note that homogeneous oxide glasses only exhibit positive values of *ν*, although negative *ν* is possible in other material families [6]. As such, *ν* is also closely related to the atomic packing density (*C*_g_) [7], with compressible silica having a low *C*_g_ and thus a low *ν*. When subjected to high pressure, silica becomes densified (hence, larger *C*_g_) and the Poisson’s ratio increases [8]. 

Another interesting proposed correlation is that between *ν* and fracture energy, with an abrupt brittle-to-ductile transition at *ν* = 0.32 for various glassy systems [7,9,10,11]. The majority of oxide glasses exhibit *ν* < 0.32 and the search for any macroscopic ductility in oxide glasses has therefore focused on designing oxide glasses with *ν* > 0.32 [11,12]. To potentially prepare such high-*ν* glasses, it is of interest to predict the composition dependence of *ν*, as it has been possible with other glass properties, such as elastic modulus (*E*) [13], hardness [14], and liquid fragility (*m*) [15]. Based on the positive correlation between Poisson’s ratio and increasing atomic packing density [7,10,11], Makishima and Mackenzie [16] established a model that, however, underestimates the Poisson’s ratio of borate and phosphate glasses and overestimates it for germanate and aluminate glasses. Molecular dynamics simulations typically also fail, including an attempt on alkali silicate glasses, for which the error in prediction of Poisson’s ratio (~20%) is much higher than that in Young’s and shear moduli (~10%) [17]. An alternative approach would be to predict a surrogate for *ν*. One candidate is the liquid fragility that has been suggested to correlate to the Poisson’s ratio [18], as the former can be predicted based on network rigidity [15,19]. However, the *m*-*ν* relation has been questioned [7,11,20], although positive correlations are observed within narrow compositional regions such as alkali silicates [10]. 

Glass structure has also been correlated with the Poisson’s ratio, as an increase in connectivity (increasing number of bridging oxygens (*n*_BO_)) results in a decrease in Poisson’s ratio [7]. This can be understood because a highly cross-linked network has more space between atoms to be compressed by changing the bond length or bond angle. In contrast, highly modified glasses, and thus, less cross-linked glasses, have less voids in the structure as these are occupied by modifiers. For example, silica with *n*_BO_ = 4 and a heavily modified silicate glass with *n*_BO_ = 2 exhibit low and high *ν*, respectively. In contrast, binary alkali borate glasses with heavy alkali ions show an overall increase in Poisson’s ratio with increasing alkali content [21], and thus, Poisson’s ratio increases with *n*_BO_. As such, the structures of borate and silicate glasses evolve differently upon modifier addition, causing differences in *n*_BO_ due to changes in both short- and medium-range structure. Boron units change between trigonal (B^3^) and tetrahedral (B^4^) coordination states, with B^4^ increasing with increasing modifier content until reaching a maximum value (around 30–40 mol% modifier oxide) where non-bridging oxygens (NBOs) start to form. The change from B^3^ to B^4^ increases the connectivity of the network. In contrast, silicon units only occur in tetrahedral coordination (*Q*^n^ where n is the number of bridging oxygens) and the addition of modifiers results in NBO formation, and thus decreasing connectivity. The structure of binary alkali silicate glasses is reported in literature [22,23] showing an approximately linear decrease in *Q*^4^ (and similar increase in *Q*^3^) with increasing alkali content up to ~35 mol%, where, *Q*^2^, *Q*^1^, and *Q*^0^ species start to form. Another important difference between the borate and silicate glasses is the superstructures. The silicate network is organized in rings, usually around 6–7-membered rings in pure silica and larger sized rings in alkali silicate glasses [24]. Borate glasses feature a rich variation of superstructural units, from boroxol rings in pure boron oxide to pentaborates, diborates, metaborate, non-ring BO_4_ units, pyroborate, and orthoborate units [25,26,27]. 

Different alkali and alkaline earth oxides have been used to modify glass structures. Their difference in size and charge results in different charge distribution as described by the modified field strength (*FS*), which is calculated from the charge (*z*) and ionic radius (*r*) as *FS* = *z*_alkali_/(*r*_alkali_ + *r*_oxygen_)^2^ [28]. *FS* is strongly correlated with various mechanical properties, including hardness [12,29,30] and elastic moduli [12,17,31], but also glass transition temperature [12,29,32,33]. Interestingly, as the higher field strength of small alkali ions like lithium compared to rubidium and cesium causes a higher atomic packing in the former, it should be expected that Poisson’s ratio is higher for lithium relative to cesium containing glasses (due to the *C*_g_-*ν* correlation [7,10,11]). However, this is not the case as glasses with larger alkali ions generally exhibit larger *ν*, both in experimental and simulation studies [12,17]. 

In this paper, we attempt to further understand the variation in Poisson’s ratio of binary cesium borate and cesium silicate glasses with varying modifier content. The silicate and borate connectivity decreases and increases, respectively, within increasing modifier content and are thus interesting to compare. Cesium is chosen as the modifier as it results in higher *ν* compared to the smaller alkali modifiers. For example, Cs_2_O-5SiO_2_ glass has been reported to have high *ν* (0.357) [34], and also cesium borates at high cesium content show high *ν* (up to 0.34 for 39 mol% Cs_2_O) [21]. The two glass series also enable us to further test different correlations proposed in literature between *ν* and various features on simple oxide glass systems. This is currently not possible based only on literature data as, e.g., the liquid fragility data is missing for both series and other properties are reported for only one cesium silicate glass. Thus, we synthesize a cesium silicate series to investigate how higher and lower cesium content will affect *ν*. We obtain structural information of the network forming species using a statistical mechanics model as structural information of the exact compositions investigated in this study is not available. We also extend the review of the network connectivity dependence of the Poisson’s ratio by including data for borate and phosphate glasses, which are missing in the original study [7].

## 2. Materials and Methods 

### 2.1. Sample Preparation 

Cesium borate and cesium silicate glasses were prepared in the following series: *x*Cs_2_O-(100−*x*)B_2_O_3_ with *x* = 10, 15, 20, 25, and 30, and *y*Cs_2_O-(100−*y*)SiO_2_ with *y* = 14, 16.7, 20, 25, and 30. This was done by first mixing Cs_2_CO_3_ (Sigma-Aldrich, 99.9%, Steinheim, Germany) with H_3_BO_3_ (Sigma-Aldrich, ≥99.5%, Steinheim, Germany) or SiO_2_ (Merck, ≥99.5%, Darmstadt, Germany) in appropriate amounts. The borates and silicates were melted in Pt crucibles at 900–1100 °C and 1600–1675 °C, respectively. The homogenized melts were quenched onto a brass plate and annealed for 30 min at their estimated glass transition temperature (*T*_g_). The low-Cs containing silicate glasses were quenched, crushed, remelted, and finally quenched again to obtain bubble-free glasses. The glasses were post-annealed at their actual *T*_g_ (see Table 1) as determined by differential scanning calorimetry (DSC, see below). We note that the measured *T*_g_ and density of the present glasses are within 3% of the values reported previously [35]. To limit surface hydration, the samples were stored in desiccators.

### 2.2. Characterization

*T*_g_ was determined by DSC using a STA 449C Jupiter instrument (Netzsch, Selb, Germany). The samples were heated in a Pt crucible in argon atmosphere above their *T*_g_ at 30 °C min^−1^ followed by cooling at 10 °C min^−1^. A second upscan was then carried out at 10 °C min^−1^ to determine *T*_g_ with a heating rate similar to the former cooling rate. *T*_g_ was determined within ±3 °C of the viscometric *T*_g_ determined as the isokom temperature at 10^12^ Pa s [36]. Angell’s liquid fragility index (*m*) was also determined using DSC with heating/cooling rates of 5, 10, 20, and 30 °C min^−1^, where the heating rate equals the previous cooling rate. The fragilities were corrected for a systematic error using Equation (1) [37].

(1) $$m=1.289\left(m_{\mathrm{DSC}}-m_{0}\right)+m_{0}$$

Here, *m*, *m*_DSC_, and *m*_0_ are the liquid fragility determined from viscosity, the liquid fragility determined from DSC, and the fragility of a perfectly strong glass that equals 14.97, respectively.

Density (*ρ*) was determined by Archimedes’ principle using ethanol as immersion medium. Each sample was weighed ten times in air and ethanol. From the density and chemical composition, we calculated the molar volume (*V*_M_) and atomic packing density (*C*_g_) using Equations (2) and (3).

(2) $$V_{M}=\frac{1}{\rho }\sum_{i}^{\;}x_{i}M_{i}$$

(3) $$C_{g}=\frac{1}{V_{m}}\sum_{i}^{\;}x_{i}V_{i}$$

Here *x*_i_, *M*_i_, and *V*_i_ are the mole fraction, molar mass, and ionic volume, respectively, of each compound. Structural assumptions of network forming cations (silicon *Q*^2^, *Q*^3^, and *Q*^4^ and boron B^3^ and B^4^) are based on statistical mechanics predictions (see, e.g., Ref. [38]) using NMR data from Dupree et al. [22] and Zhong and Bray [39], while cesium is expected to be six-fold coordinated. The ionic radii of cesium, boron, silicon, and oxygen are taken from the work of Shannon [40].

For characterization of elastic properties, the samples were ground using SiC paper in ethanol to obtain coplanar surfaces. The longitudinal and transverse wave velocities (*V*_L_ and *V*_T_, respectively) were measured by an ultrasonic thickness gauge (38DL Plus, Olympus, Tokyo, Japan) using the pulse-echo method with 20 MHz delay line. The thickness of the samples were measured with a digital micrometer with a precision of 0.01 mm. *V*_L_ and *V*_T_ were calculated based on the time between the initial pulse and the echo. Poisson’s ratio (*ν*) was calculated from *V*_L_ and *V*_T_, following Equation (4). The wave velocities were measured just after polishing to avoid hydration effects.

(4) $$\nu =\frac{V_{L}^{2}-2V_{T}^{2}}{2\left(V_{L}^{2}-V_{T}^{2}\right)}$$

The shear (*G*) and elastic (*E*) moduli were calculated from Equations (5) and (6), respectively.

(5) $$G=\rho \;V_{T}^{2}$$

(6) E=2G(1+ν)

## 3. Results

**Figure 1:**
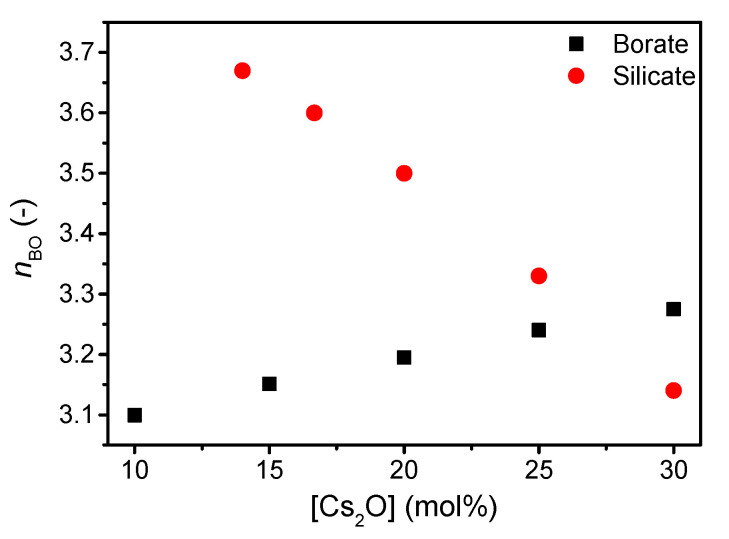
Number of bridging oxygens (*n*_BO_) in silicate and borate network with increasing cesium content. Values are based on statistical mechanics modelling from Supplementary Figures S1 and S2.

The structure of the present silicate and borate glasses are predicted using a statistical mechanics model [38] based on existing ^29^Si and ^11^B magic angle spinning (MAS) NMR spectroscopy data for other compositions [20,39]. The silicate network becomes increasingly depolymerized with increasing cesium content visible by the decrease in *Q*^4^ species (Supplementary Figure S1) and, thus, an increase in *Q*^3^ species. In contrast to the depolymerizing silicate network, the borate network features a higher degree of connectivity as B^3^ units are replaced by B^4^ units with increasing cesium content (Supplementary Figure S2). The change in connectivity is reflected in the calculated change in average number of bridging oxygens (*n*_BO_) (Figure 1).

The measured properties (*T*_g_, *m*, *ρ*, *C*_g_, *E*, *G*, and *ν*) of the cesium silicate and borate glasses are summarized in Table 1. The addition of cesium oxide to the borate and silicate glasses results in an increase and decrease in elastic moduli, respectively. The shear modulus (*G*) of both the borate and silicate series is similar to previously reported values [21,41]. For the borate series, the moduli initially increase, followed by a minor decrease, and finally increase with increasing cesium content. This non-monotonic variation has been ascribed to the changes in superstructural units [42]. For both series, *m* is found to increase with increasing cesium content. In silicate glasses, the increase is due to the depolymerization of the network. In contrast, the borate network is becoming increasingly polymerized when adding modifiers, but again the variation in intermediate-range order (superstructural units) results in an increased fragility with increasing modifier content [43]. 

The glass transition temperature exhibits the same compositional trends as the elastic moduli for both the borate and the silicate series, i.e., an overall decrease and increase in *T*_g_ for silicate and borate glasses with increasing cesium content, respectively (Table 1). However, for the borate series, the *T*_g_ decreases at the highest cesium content (30 mol%), presumably due to the initial formation of NBOs that occurs at lower alkali content for large alkali ions like cesium [39]. The decrease in *T*_g_ of the silicate network is due to the replacement of strong Si-O bonds by weaker Cs-O bonds and the formation of NBOs, whereas the increase in *T*_g_ of the borate network is due to the increase in network connectivity. The large changes in network structure lead to relatively large changes in *T*_g_ (up to ~85 °C and ~55 °C for borate and silicate series, respectively), in agreement with previously reported data [44,45,46].

**Figure 2:**
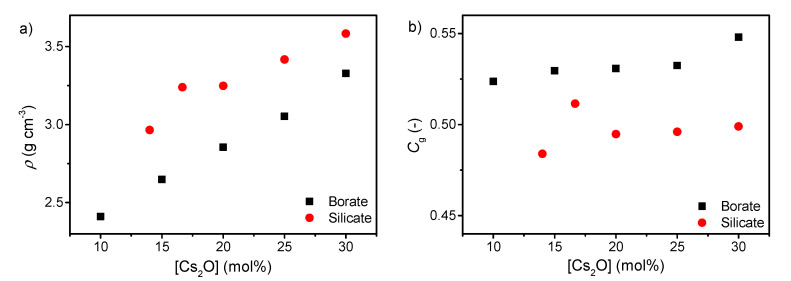
Dependence of (**a**) density (*ρ*) and (**b**) atomic packing density (*C*_g_) on cesium content in binary borate and silicate glasses. The errors in *ρ* and *C*_g_ are estimated to be 0.01 g cm^−3^ and 0.002, respectively.

The density of both silicates and borates increases with increasing cesium content (Figure 2a), consistent with the higher molar mass of cesium oxide compared to boron oxide and silica. This is in agreement with results for various binary alkali borates and silicates [47,48,49]. The atomic packing density also increases with increasing cesium content as well (Figure 2b). This is due to the more tightly packed network when the cesium ions occupy the interstitial sites in order to charge balance the tetrahedral borate species and the formed NBOs in the silicate network. It has previously been shown that a larger fraction of B^4^ results in a larger atomic packing density [50], in agreement with these results. The lower *C*_g_ of the silicate series compared to the borate series is consistent with the more open microstructure of pure SiO_2_ glass compared to B_2_O_3_ glass.

**Figure 3:**
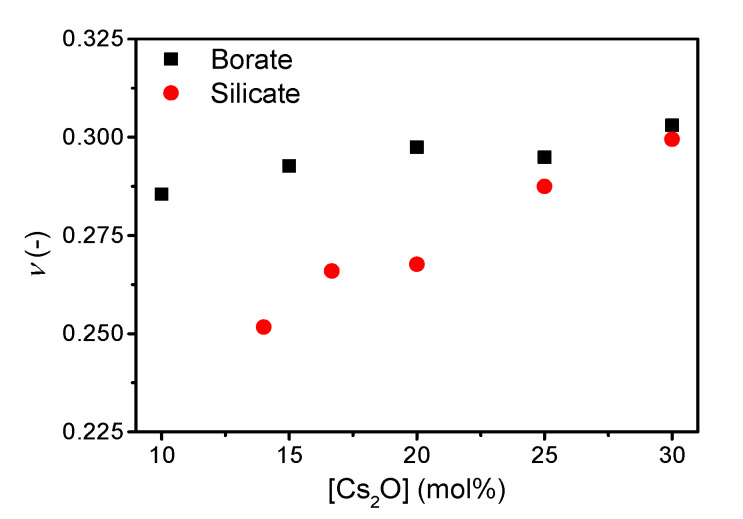
Poisson’s ratio (*ν*) as a function of cesium content in binary borate and silicate glasses. The error in *ν* is estimated to be 0.01.

The addition of cesium oxide to the borate and silicate network increases the Poisson’s ratio (Figure 3), with *ν* ranging from 0.286 to 0.303 and 0.252 to 0.299 for the borate and silicate glass series, respectively. The trend in Poisson’s ratio with increasing cesium content in the borate series is similar to that previously reported [21] with an increase towards 20 mol%, a small decrease up to 25 mol%, followed by an increase at higher cesium content.

## 4. Discussion

Poisson’s ratio has been suggested to be correlated to liquid fragility [18], densification [5], network connectivity [7], and fracture energy [9]. In the following, we discuss the effect of changes in structure and liquid fragility on Poisson’s ratio, which has been found to increase with increasing cesium content in the binary borate and silicate glasses (Figure 3). The link of Poisson’s ratio with liquid fragility was originally proposed by Novikov and Sokolov [18]. However, the linear correlation does not exist across various glass families [7,11,20]. Here, considering compositionally simple oxide glasses, we do observe a positive linear correlation between *ν* and *m* in cesium silicate and borate glasses (Supplementary Figure S3), as previously shown for sodium and potassium silicate glasses [10]. The correlation is stronger in the silicate system, possibly due to the larger range of Poisson’s ratio values. However, the *ν* vs. *m* slope is not universal.

**Figure 4:**
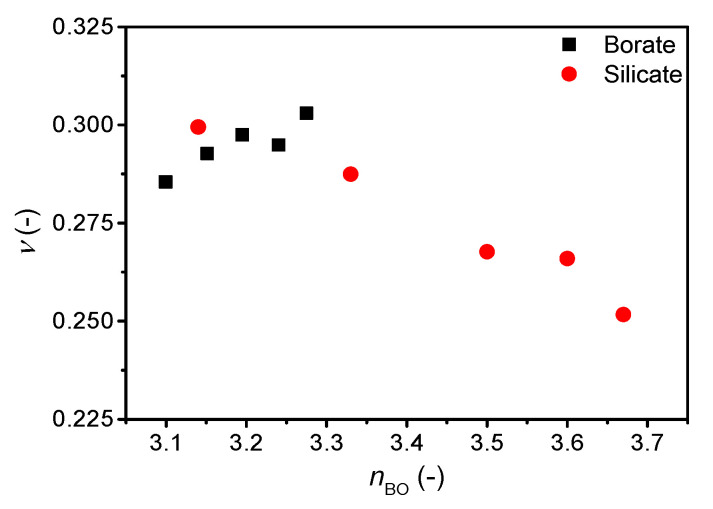
Dependence of Poisson’s ratio (*ν*) on average number of bridging oxygens (*n*_BO_) in binary cesium borate and silicate glasses. The error in *ν* is estimated to be 0.01.

The correlation of Poisson’s ratio with the atomic packing density is shown in Supplementary Figure S4 and that with the average number of bridging oxygens (*n*_BO_) is shown in Figure 4. We find an increase in Poisson’s ratio with increasing atomic packing density (Supplementary Figure S4), but similarly to the *ν*-*m* correlation, the *ν* vs. *C*_g_ slope varies among systems. The change in *C*_g_ is relatively small, but as shown previously, a broad range of Poisson’s ratios are found around *C*_g_ = 0.5 [7,11]. In contrast, the change in Poisson’s ratio with *n*_BO_ is opposite for cesium silicate and borate glasses. In the silicate glasses, the network connectivity decreases with increasing cesium content due to formation of NBOs in the structure, causing an increase in Poisson’s ratio as previously described [7]. Consequently, the increasing average coordination number of boron (increasing connectivity) with the increasing cesium content should cause a decrease in Poisson’s ratio, but the opposite is observed (Figure 4). The change in Poisson’s ratio with composition is minor (Figure 3), showing a small increase from 10 to 20 mol% and then the value of Poisson’s ratio appears to be constant between 20 and 30 mol%. The previous study [21] ascribed the lack of increase in Poisson’s ratio in this region to borate superstructural units, especially the formation of pentaborate units. Therefore, the connectivity might not be sufficiently captured by the short-range order metric *n*_BO_ in borate glasses as various superstructural units are present throughout the compositional range.

**Figure 5:**
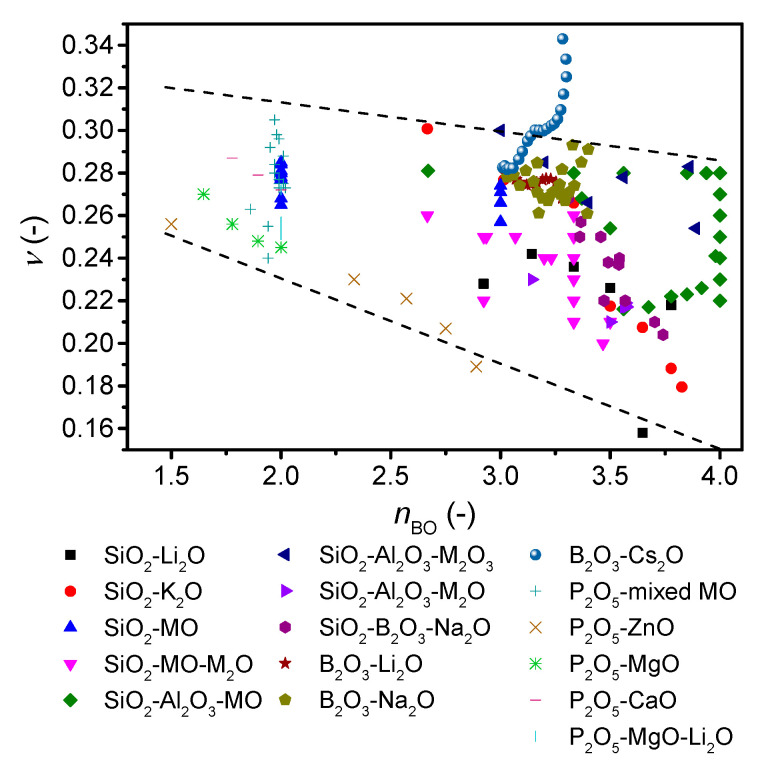
Dependence of Poisson’s ratio (*ν*) on average number of bridging oxygens (*n*_BO_) of various oxide glass forming systems. The error in *ν* is estimated to be 0.01. The dashed lines are guides for the eye, showing the trends for the majority of the data.

To further investigate the role of network connectivity on Poisson’s ratio, we revisit the *ν*-*n*_BO_ correlation by replotting Figure 4 with literature values for a variety of alkali and alkaline earth silicate [45,49,51,52,53], borosilicate [50,54,55], aluminosilicate [30,50,56,57], alkali borate [42,58,59,60], and phosphate glasses [61,62,63,64] (Figure 5). Here, the boron and phosphorus containing glasses are new compared to the original study [7]. The number of bridging oxygens is either taken directly from the reference, calculated from NMR data (*Q*^n^ or *B*^3/4^ units) if available (these describe whether the oxygen bonded to a Si unit or B unit is bonded to another Si/B or is an ionic oxygen charge balanced by modifier ions), predicted from statistical modelling (as described in Figure 1 and Supplementary Figures S1 and S2) [38,65], or estimated as Equation (7),

(7) $$n_{\mathrm{BO}}=\frac{\sum_{i}M_{i}z_{i}}{\sum_{j}F_{j}}$$

where *M*, *z*, and *F* are the atomic fraction of the *i*th modifying cation (after deduction of the number of charge compensators), the valence of the *i*th modifying cation, and the fraction of the *j*th network forming cation (for details, see Ref. [7]). Considering first the overall trend in Figure 5, a negative correlation between Poisson’s ratio and *n*_BO_ is seen as expected [7], but the range of Poisson’s ratios at each *n*_BO_ value is larger than that previously reported. 

**Figure 6:**
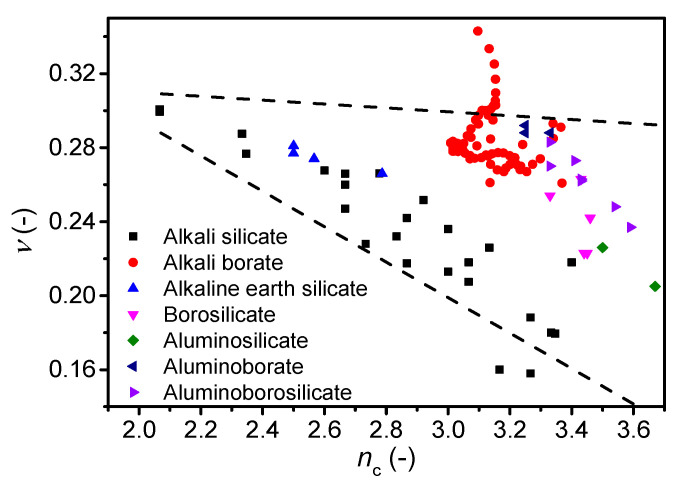
Dependence of Poisson’s ratio (*ν*) on average number of constraints per atom (*n*_c_) for various oxide glass systems. The error in *ν* is estimated to be 0.01. The dashed lines are guides for the eye, showing the trends for the majority of the data.

Considering the different compositions in more details, we find that the modified silicate glasses exhibit a higher Poisson’s ratio for higher modifier content and thus lower *n*_BO_. The Poisson’s ratio of aluminosilicate glasses reaches a maximum around the charge-balanced tectosilicate composition, and *ν* thus increases with increasing connectivity. However, within a broad range of calcium alumino tectosilicate glasses, *ν* increases from 0.22 to 0.28 with decreasing silica content. The calculation of *n*_BO_ is based on the assumption that all aluminum is in four-fold coordination, with no NBOs. However, there is evidence from NMR studies of up to 6% of aluminum in five-fold coordination [66] as well as up to 5% NBOs [67], and *n*_BO_ is thus not exactly four. Assuming 5% five-fold aluminum, *n*_BO_ would decrease to 3.87 and 3.95 for tectosilicate compositions with 36 mol% and 76 mol% SiO_2_, respectively. For phosphate glasses, *ν* decreases with increasing connectivity as for silicate glasses, but for the metaphosphate composition, Poisson’s ratio spans a wide range from 0.25 to 0.30. Alkali borate glasses that exhibit an increase in connectivity when modified features different trends. Lithium borates show an overall negative correlation with connectivity, whereas sodium borates show a negative trend followed by a positive trend with a minimum around 26 mol% Na_2_O. In contrast, cesium borates from literature [21] show a monotonic positive correlation with connectivity (Cs_2_O >7 mol%) and further increase in *ν* after the boron anomaly as also shown in Figure 6, although with a plateau in *ν* around 17–25 mol%. This variation in trend for borate glasses can be ascribed to the various types of superstructural units, as the minima/plateau region is explained by formation of pentaborate units [21]. Increasing the alkali content above 25 mol% results in reformation of pentaborate units to diborate, metaborate, and non-ring borate units, causing a looser structure, and therefore an increase in Poisson’s ratio. The size of the cation also plays a significant role, as only larger cations (potassium, rubidium, and cesium) show an overall increase in Poisson with increasing alkali content [21]. For mixed network-former borosilicate glasses, a negative *ν*-*n*_BO_ correlation is observed. This is probably due to the limited amount of borate superstructural units in high-silica borosilicate glasses as the borate units are mixed with the silicate network, with Si-O-B bonding [68]. Overall, we conclude that the negative correlation between Poisson’s ratio and *n*_BO_ does not apply to all oxide glass systems.

Finally, we investigate the correlation between *ν* and another metric for network connectivity, namely the number of constraints per atom (*n*_c_) as calculated from topological constraint theory (TCT). TCT has been used to predict other characteristics of oxide glasses, such as *T*_g_, *E*, dissolution rate, hardness, and liquid fragility [13,14,69,70,71]. The theory was originally developed by Phillips and Thorpe [72,73], and later extended to account for temperature-dependent constraints [70]. Each atom in the glass has a number of constraints that can be divided into bond-bending and bond-stretching, which in turn can be calculated from the atomic coordination numbers [74]. We test the correlation for the glasses in this study as well as glasses from literature, namely alkali silicates [17,45,49], alkali borates [42,58,59,60], alkaline earth silicates [51], and borosilicates, aluminosilicates, aluminoborates, and aluminoborosilicates [50]. The number of constraints are calculated differently. For alkali and alkaline earth silicate glasses, we use equations proposed by Micoulaut [75], while for the remaining glasses the number of constraints are calculated using Equation (8) (see Ref. [76] for details) based on structural data (fraction of species, *N*) obtained by either statistical mechanics or NMR data.

(8) $$n_{c}=3N\left(O\right)+5N\left(\mathrm{Si}\right)+7N\left({\mathrm{Al}}^{V}\right)+5N\left({\mathrm{Al}}^{\mathrm{IV}}\right)+5N\left(B^{\mathrm{IV}}\right)+3N\left(B^{\mathrm{III}}\right)+2N\left(M-\mathrm{NBO}\right)$$

In contrast to the *n*_BO_ calculation, one can account for the superstructural units in lithium borate glasses using TCT [77]. However, the correlation between *ν* and *n*_c_ when using TCT with and without accounting for superstructural units is similar, although the range of constraints is minimized when accounting for superstructural units (Supplementary Figure S5). In general, the Poisson’s ratio is found to decrease with an increasing number of constraints for all glass families (Figure 6). However, as for the number of bridging oxygens (Figure 5), the cesium borate glasses show an opposing trend as *ν* increases with *n*_c_. These findings on cesium borate glasses combined with the studies on alkali borate glasses in literature [21,42] suggest that more work is needed to understand the effect of superstructural units on Poisson’s ratio. 

## 5. Conclusions

We have investigated how the Poisson’s ratio of binary cesium silicate and cesium borate glasses varies with the underlying network structure. We find an increase in atomic packing density with cesium content for both series, while the glass transition temperature decreases for the silicate but increases in the borate series. Poisson’s ratio increases in both series with cesium content, liquid fragility, and atomic packing density. With respect to the role of network connectivity, we find that the borate and silicate series show a positive and negative correlation with connectivity, respectively. A similar correlation is found between Poisson’s ratio and the number of constraints per atom.

## Figures and Tables

**Table 1:** Nominal chemical composition, glass transition temperature (*T*_g_), liquid fragility (*m*), density (*ρ*), atomic packing density (*C*_g_), Young’s modulus (*E)*, shear modulus (*G*), and Poisson’s ratio (*ν*). The errors in *T*_g_, *m*, *ρ*, *C*_g_, *E*, *G*, and *ν* do not exceed 3 °C, 1, 0.01 g/cm^3^, 0.002, 2 GPa, 1 GPa, and 0.01, respectively.

Sample ID	Nominal Composition (mol%)			*T*_g_ (°C)	*M* (-)	*ρ* (g cm^−3^)	*C*_g_ (-)	*E* (GPa)	*G* (GPa)	*ν* (-)
	SiO_2_	B_2_O_3_	Cs_2_O							
Si86	86	-	14	555	20	2.97	0.484	44	18	0.25
Si83	83.3	-	16.7	549	30	3.24	0.512	40	16	0.27
Si80	80	-	20	539	30	3.25	0.495	38	15	0.27
Si75	75	-	25	530	37	3.42	0.496	33	13	0.29
Si70	70	-	30	490	47	3.58	0.499	31	12	0.30
B90	-	90	10	319	25	2.41	0.524	25	10	0.29
B85	-	85	15	343	30	2.65	0.530	26	10	0.29
B80	-	80	20	376	32	2.85	0.531	25	10	0.30
B75	-	75	25	416	47	3.05	0.532	30	11	0.30
B70	-	70	30	403	49	3.33	0.548	31	18	0.30

## Supplementary Materials

The following are available online at https://www.mdpi.com/1996-1944/13/12/2837/s1, Figure S1. Compositional dependence of the fraction of *Q*^n^ structural units in cesium. The model predictions are based on NMR spectroscopy data (squares) on cesium silicate glasses [22] and calculated as in Ref. [38]. Figure S2. Compositional dependence of the fraction of B^n^ structural units in cesium borate glasses. The model predictions are based on NMR spectroscopy data (squares) on cesium borate glasses [39] and calculated as in Ref. [38]. Figure S3. Dependence of Poisson’s ratio (*ν*) on liquid fragility (*m*) in binary cesium borate and silicate glasses. The errors in *ν* and *m* are estimated to be 0.01 and 1, respectively. Dashed lines serve as guides for the eye. Figure S4. Dependence of Poisson’s ratio (*ν*) on atomic packing density (*C*_g_) in binary cesium borate and silicate glasses. The errors in *ν* and *C*_g_ are estimated to be 0.01 and 0.002, respectively. Dashed lines serve as guides for the eye. Figure S5. Dependence of Poisson’s ratio (*ν*) on average number of constraints per atom (*n*_c_) calculated with and without taking superstructural borate units into account in the lithium borate glass. *ν* is taken from Ref. [21].
