# Supplementary material for: Structure Dependence of Poisson’s Ratio in Cesium Silicate and Borate Glasses
Source: Materials (Basel). 2020 Jun 24;13(12):2837. doi: 10.3390/ma13122837 (PMC7345141; doi:10.3390/ma13122837)
Supplement: Supplementary file 1 [file materials-13-02837-s001.pdf]

materials

MDPI

SUPPLEMENTARY MATERIALS

Structure Dependence of Poisson's Ratio in Cesium Silicate and Borate Glasses

Martin B. Østergaard, Mikkel S. Bødker and Morten M. Smedskjaer *

Department of Chemistry and Bioscience, Aalborg University, 9220 Aalborg East, Denmark
* Correspondence: mos@bio.aau.dk; Tel.: +45-9940-3578

Received: 6 May 2020; Accepted: 22 June 2020; Published: date

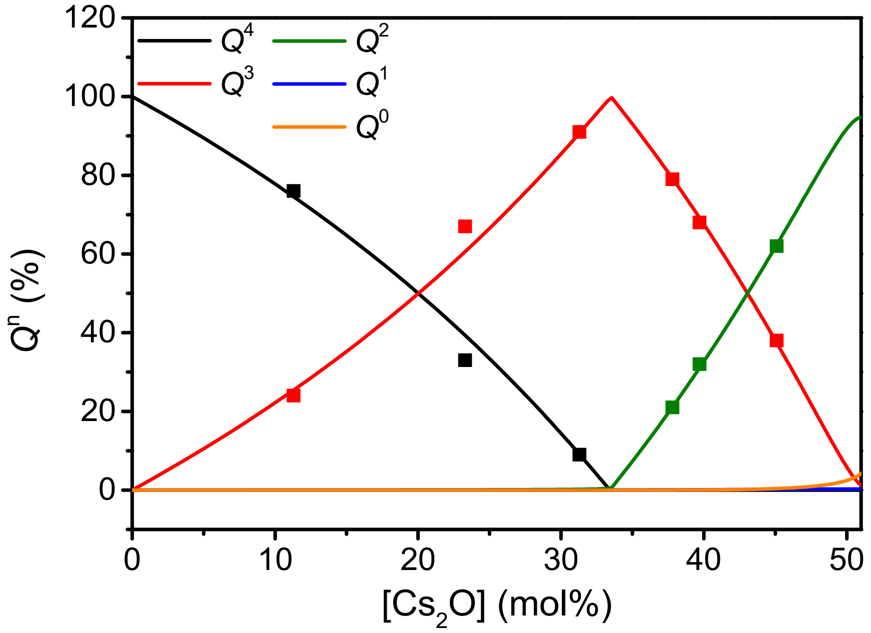

Figure S1. Compositional dependence of the fraction of Q^n structural units in cesium The model predictions are based on NMR spectroscopy data (squares) on cesium silicate glasses [1] and calculated as in Ref. [2].

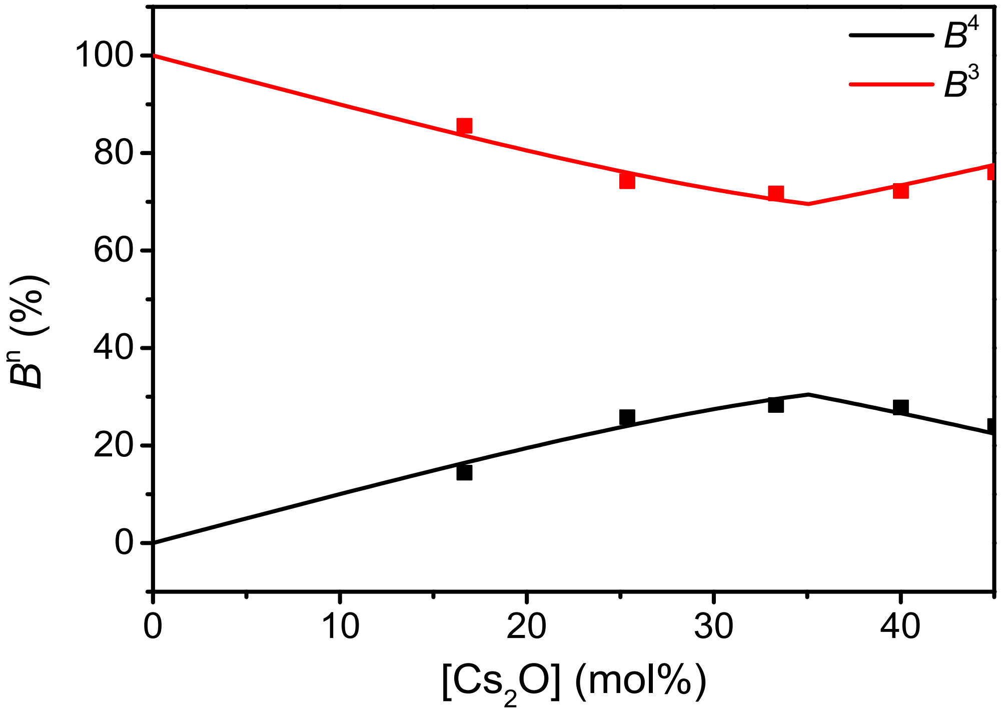

Figure S2. Compositional dependence of the fraction of B^n structural units in cesium borate glasses. The model predictions are based on NMR spectroscopy data (squares) on cesium borate glasses [3] and calculated as in Ref. [2].

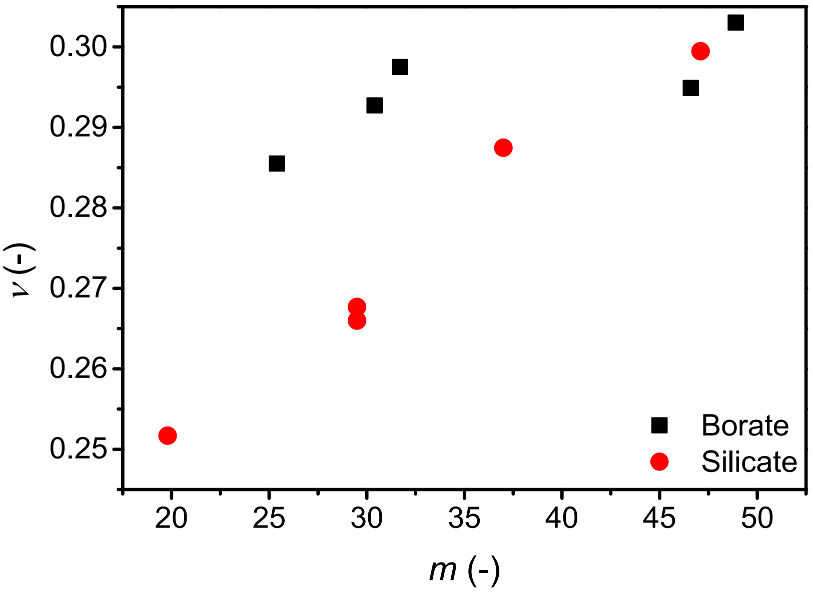

Figure S3. Dependence of Poisson's ratio (ν) on liquid fragility (m) in binary cesium borate and silicate glasses. The errors in ν and m are estimated to be 0.01 and 1, respectively.

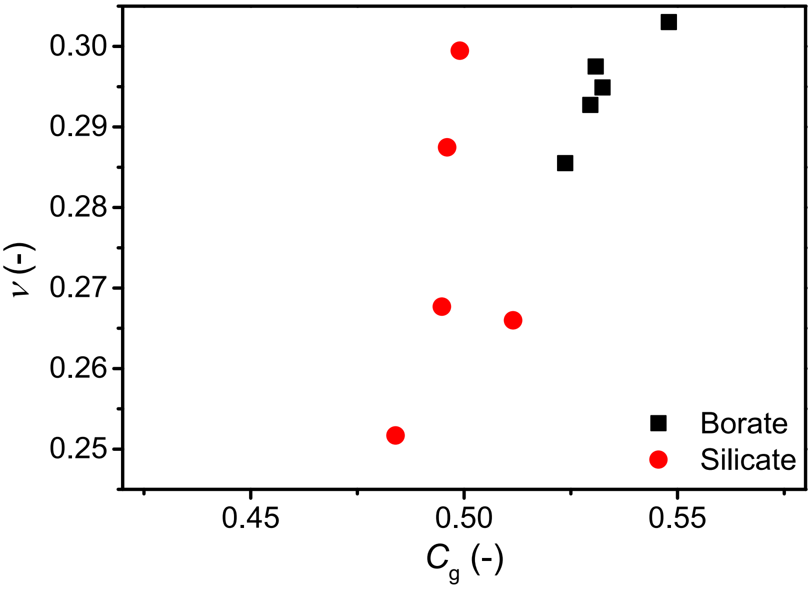

Figure S4. Dependence of Poisson's ratio (*ν*) on atomic packing density (C_g) in binary cesium borate and silicate glasses. The errors in *ν* and C_g are estimated to be 0.01 and 0.002, respectively.

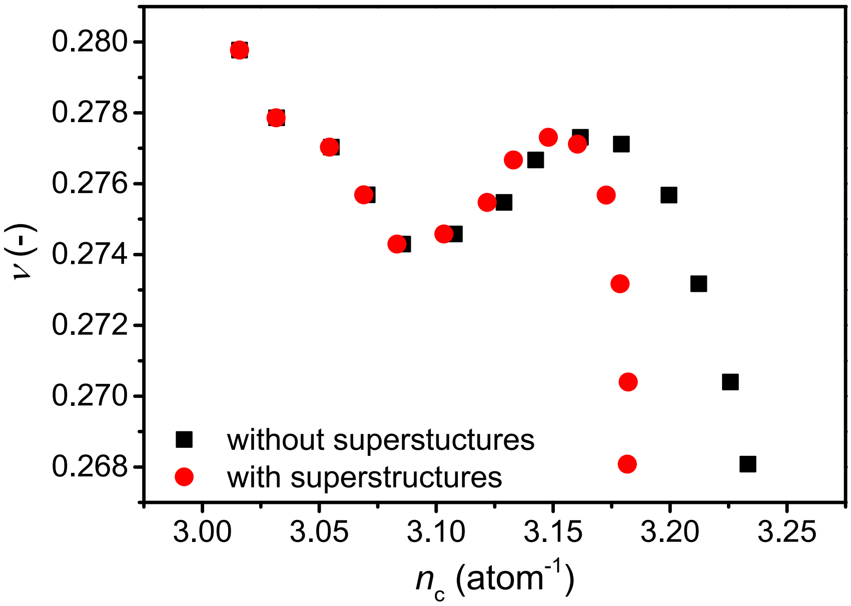

Figure S5. Dependence of Poisson's ratio (*ν*) on average number of constraints per atom (n_c) calculated with and without taking superstructural borate units into account in the lithium borate glass. *ν* is taken from Ref. [4].

References

1. R. Dupree, D. Holland, D.S. Williams, The structure of binary alkali silicate glasses, J. Non. Cryst. Solids. 81 (1986) 185–200.
2. M.S. Bødker, S.S. Sørensen, J.C. Mauro, M.M. Smedskjaer, Predicting Composition-Structure Relations in Alkali Borosilicate Glasses Using Statistical Mechanics, Front. Mater. 6 (2019) 1–11.
3. J. Zhong, P.J. Bray, Change in boron coordination in alkali borate glasses, and mixed alkali effects, as elucidated by NMR, J. Non. Cryst. Solids. 111 (1989) 67–76.
4. M. Kodama, S. Kojima, Velocity of sound in and elastic properties of alkali metal borate glasses, Phys. Chem. Glas. Eur. J. Glas. Sci. Technol. B. 55 (2014) 1–12.
